# Monocarboxylate Transporter 4 (MCT4) Knockout Mice Have Attenuated 4NQO Induced Carcinogenesis; A Role for MCT4 in Driving Oral Squamous Cell Cancer

**DOI:** 10.3389/fonc.2018.00324

**Published:** 2018-08-28

**Authors:** Sara Bisetto, Diana Whitaker-Menezes, Nicole A. Wilski, Madalina Tuluc, Joseph Curry, Tingting Zhan, Christopher M. Snyder, Ubaldo E. Martinez-Outschoorn, Nancy J. Philp

**Affiliations:** ^1^Department of Pathology, Anatomy and Cell Biology, Sydney Kimmel Cancer Center, Thomas Jefferson University, Philadelphia, PA, United States; ^2^Department of Medical Oncology, Sydney Kimmel Cancer Center, Thomas Jefferson University, Philadelphia, PA, United States; ^3^Department of Microbiology and Immunology, Sydney Kimmel Cancer Center, Thomas Jefferson University, Philadelphia, PA, United States; ^4^Department of Otolaryngology–Head and Neck Surgery, Sydney Kimmel Cancer Center, Thomas Jefferson University, Philadelphia, PA, United States; ^5^Division of Biostatistics, Department of Pharmacology and Experimental Therapeutics, Sydney Kimmel Cancer Center, Thomas Jefferson University, Philadelphia, PA, United States

**Keywords:** MCT4, 4NQO, oral squamous cell carcinoma, tumor microenvironment, metabolism

## Abstract

Head and neck squamous cell carcinoma (HNSCC) is the 6th most common human cancer and affects approximately 50,000 new patients every year in the US. The major risk factors for HNSCC are tobacco and alcohol consumption as well as oncogenic HPV infections. Despite advances in therapy, the overall survival rate for all-comers is only 50%. Understanding the biology of HNSCC is crucial to identifying new biomarkers, implementing early diagnostic approaches and developing novel therapies. As in several other cancers, HNSCC expresses elevated levels of MCT4, a member of the SLC16 family of monocarboxylate transporters. MCT4 is a H^+^-linked lactate transporter which functions to facilitate lactate efflux from highly glycolytic cells. High MCT4 levels in HNSCC have been associated with poor prognosis, but the role of MCT4 in the development and progression of this cancer is still poorly understood. In this study, we used 4-nitroquinoline-1-oxide (4NQO) to induce oral cancer in MCT4^−/−^ and wild type littermates, recapitulating the disease progression in humans. Histological analysis of mouse tongues after 23 weeks of 4NQO treatment showed that MCT4^−/−^ mice developed significantly fewer and less extended invasive lesions than wild type. In mice, as in human samples, MCT4 was not expressed in normal oral mucosa but was detected in the transformed epithelium. In the 4NQO treated mice we detected MCT4 in foci of the basal layer undergoing transformation, and progressively in areas of carcinoma *in situ* and invasive carcinomas. Moreover, we found MCT4 positive macrophages within the tumor and in the stroma surrounding the lesions in both human samples of HNSCC and in the 4NQO treated animals. The results of our studies showed that MCT4 could be used as an early diagnostic biomarker of HNSCC. Our finding with the MCT4^−/−^ mice suggest MCT4 is a driver of progression to oral squamous cell cancer and MCT4 inhibitors could have clinical benefits for preventing invasive HNSCC.

## Introduction

Head and neck squamous cell carcinoma (HNSCC) is the 6th most common human cancer with almost 50,000 new cases diagnosed in the United States each year (1, 2). Among the different subtypes of HNSCC, oral squamous cell carcinoma (OSCC), especially of the tongue, is the most common. Risk factors for OSCC are tobacco use and alcohol consumption (3, 4). The high mortality with HNSCC and OSCC in particular, with a 5-year survival rate of only 50%, creates an urgent need for better understanding of disease biology as well as prognostic and predictive biomarkers and novel therapies.

Recent studies have shown that, similar to other types of cancer, an increase in monocarboxylate transporter 4 (MCT4) expression in patients with OSCC correlates with a poor outcome (5–8). MCT4 is a member of the SLC16 family of solute transporters and functions as a proton-coupled lactate transporter (9). MCT4 is primarily expressed in glycolytic cells including fast twitch muscle, neural retina and activated macrophages where it facilitates the efflux of lactate (10–12). Warburg was the first to show that cancer cells often exhibit a high rate of aerobic glycolysis producing large amounts of lactate. In tumors, high levels of lactate are generated by a subset of highly glycolytic cancer and stromal cells. It is then taken up and oxidized by other tumor cells expressing high levels of monocarboxylate transporter 1 (MCT1), another member of the SLC16 family (13). Lactate is metabolized through oxidative phosphorylation to produce ATP and intermediates that support tumor growth. In addition to its role as metabolic substrate, lactate is also a signaling molecule with important roles in angiogenesis, tumor migration and invasion, as well as modulation of the immune system (14).

The experimental models currently available for understanding the contribution of MCTs and lactate to the progression of cancer are based on the manipulation of gene expression in cancer cell lines for *in vitro* and *in vivo* studies (15–17). These studies have contributed to a greater understanding of the role of lactate in tumor progression and survival, highlighting the therapeutic potential of targeting MCT1 and MCT4. However, the importance of epithelial and stromal MCT4 in driving cancer progression remains poorly understood.

In this study we investigated the role of MCT4 in the progression of OSCC in a well-established model of oral squamous cell carcinoma using the carcinogen 4-nitroquinoline-1-oxide (4NQO) (18) in wild type (MCT4^+/+^) and MCT4 knockout (MCT4^−/−^) mice. After exposure to 4NQO, MCT4 knockout animals developed significantly fewer and less extensive invasive SCC lesions compared to wild type mice. Importantly, MCT4, which is typically absent in normal tongue epithelium, was expressed early in regions of dysplastic epithelium and later in areas of *in situ* carcinomas (CIS) and invasive squamous cell carcinomas (SCC). In addition, MCT4 was detected in macrophages within the lesion and adjacent stroma after 4NQO exposure, similar to what is observed in human OSCC samples. Our results suggest that MCT4 is critical for the progression from dysplastic lesions to invasive cancer and is therefore a relevant therapeutic target for the treatment of OSCC.

## Materials and methods

### Human study

This study was approved by the institutional review board (IRB) at Thomas Jefferson University. Samples of primary tumors from 9 patients with head and neck cancer were obtained from archived paraffin-embedded tissue blocks for histological analysis. Patient data were collected, including: age, sex, tobacco use, stage of disease, location of tumor, and histological features.

### Animals

MCT4^+/−^ mice were purchased from Taconic Bioscience. The animals were backcrossed for 10 generations to C57Bl/6N (Taconic) mice and MCT4^+/−^ mice were used for breeding to obtain knock out and wild type littermates. Genotype was confirmed by PCR. Mice were kept in a 12:12 light/dark cycle and provided with *ad libitum* food and drinking water.

### Mouse oral carcinogenesis induction

MCT4^−/−^ and wild type mice (*n* = 15–16) 12 weeks of age, were treated with 4-nitroquinoline-1-oxide (4NQO; cat # N8141, Sigma-Aldrich) in the drinking water at a concentration of 50 μg/ml. The animals were treated for 16 weeks with the 4NQO and then for an additional 7 weeks with water only. Fresh 4NQO/water was supplied every week. Animals were sacrificed after 14 weeks of treatment and at 23 weeks or when body weight loss was >20% of original weight. Oral cavities were inspected weekly for signs of lesions, and body weight was monitored as a sign of distress. All the experiments were conducted in accordance and with the approval of the Institutional Animal Care and Use Committee (IACUC) at Thomas Jefferson University.

### Antibodies

The following antibodies were used: MCT4 (SLC16A3) 19-mer peptide sequence CKAEPEKNGEVVHTPETSV-cooh affinity purified rabbit antibody and MCT1 (SLC16A1) 19-mer peptide sequence CSPDQKDTEGGPKEEESPV-cooh affinity purified rabbit antibodies were generated by YenZym Antibodies, South San Francisco, CA. (11). Mouse anti-human MCT4 (D-1) antibody was from Santa Cruz Biotechnology. Rabbit anti human- CD163 was from Abcam. Rat anti-mouse F4/80 (CI-A3-1) was from Novus Biologicals. CD45.2 (clone 104), PD-L1 (clone 10F.9G2), Ly6C (clone HK1.4), Ly6G (clone 1A8), CD11b (clone M1/70) were from BioLegend.

### Analysis of human dual labeling for CD163-positive macrophages and MCT4

Paraffin sections of human HNSCC were dual labeled by immunohistochemistry as detailed below with CD163 (Abcam) developed in red/pink and MCT4 (Santa Cruz Biotechnology) developed in brown. Slides were scanned at low power (4x) and 3 areas containing cancer cells and identifiable macrophages (stained in red/pink) that were within tumor nests or in the immediate adjacent stroma were selected and then scored at high power (40x). Each 40x field was scored as positive when >80% macrophages were also MCT4 positive. The same method was used to evaluate normal, non-tumor areas within the same section. These areas were located in stroma underlying normal squamous epithelium or in areas of non-involved muscle.

### Macroscopic photography and lesional area quantification

Tongues were harvested and placed on ice in phosphate buffered saline (PBS). Immediately prior to photography, individual tongues were incubated for one minute in a solution of 10% acetic acid and 4% ethanol followed by two PBS rinses [modified from Mashberg et al. (19)]. This treatment accentuated lesional areas from relatively unaffected areas. Images were captured with AxioVision LE software using a 5megapixel Zeiss AxioCam Erc5s color camera coupled to a Stemi 2000C Zeiss stereomicroscope at 0.65x magnification. ImageJ was used for the quantification of the affected areas on the dorsal surface of the tongue. Briefly, lesional areas which were identified as white opaque raised regions, were measured and the average percentage lesional area was calculated for wild type versus MCT4^−/−^ tongues. The images were evaluated in a blinded manner by 2 independent observers.

### Tissue collection and histopathological analysis

Tongue samples were fixed in 10% formalin and then cut lengthwise into 3 pieces and embedded separately in paraffin. Four micron sections were cut from all blocks and stained with hematoxylin and eosin (H&E) for histopathological evaluation by an expert in oral cancer pathology, to determine presence of carcinoma *-in-situ* and invasive carcinoma. In other experiments, tongues samples were cut lengthwise and either flash frozen in liquid nitrogen or embedded in OCT compound for frozen sectioning.

### Immunohistochemistry of paraffin and frozen sections

A 3-step avidin-biotin horseradish peroxidase method was used for single antibody labeling on paraffin sections as previously described (20). Briefly after deparaffinization and rehydration of the sections, antigen retrieval was performed in 0.01M citrate buffer, pH 6.0 using an electric pressure cooker. An avidin-biotin kit (Biocare Medical) was used to block endogenous biotin and the sections were blocked with 10% goat serum (Vector Labs) in PBS at 4°C. Primary antibody was incubated for 1 hour followed by biotinylated species-specific secondary antibody (Vector Labs) and avidin-biotin-horseradish peroxidase complex (ABC Elite Kit, Vector Labs) with washing in PBS between steps. Antibody binding was detected with 3,3′ diaminobenzidine (DAB liquid substrate kit, Agilent Technologies). A similar procedure was used for frozen sections that were fixed in acetone. For dual antibody labeling, a 2-step procedure was used as described in (21). Briefly, after antigen retrieval and blocking in 5% BSA, sections were incubated simultaneously with primary antibodies followed species-specific secondary antibodies conjugated with alkaline phosphatase or peroxidase (Jackson ImmunoResearch). Antibody binding was detected using DAB liquid substrate kit and ImmPACT Vector Red substrate kit (Vector) containing 1.25 mM levamisole (Sigma-Aldrich). Light microscopy images were captured using cellSens Entry software and the Olympus DP22 camera attached to an Olympus CX41 microscope (Olympus Scientific Solutions Americas).

### Dual labeling by immunofluorescence

For mouse samples double labeling immunofluorescence was performed using frozen sections after fixation in acetone and blocking in 5% BSA. Primary antibodies were incubated together for one hour at RT, washed and detected with AlexaFluor 488 conjugated donkey anti-rat IgG and AlexaFluor 568 conjugated goat anti-rabbit IgG (ThermoFisher Scientific). Nuclei were stained with DAPI and sections were mounted with ProLong Gold anti-fade (ThermoFisher Scientific). All immunofluorescent labeling images were captured on the Nikon A1R confocal microscope with a 40x oil objective lens with or without 2x zoom.

### Macrophage quantification in 4NQO-treated mice

Macrophages were identified by immunohistochemistry using the F4/80 antibody in frozen tissue sections at 14 and 23 weeks. Areas of involved and non-involved epithelia were identified by a pathologist with expertise in head and neck pathology, and 3–5 digital images were taken at 20× magnification and analyzed with Image J software calibrated for magnification. The subepithelial stromal area for each image was measured and the number of F4/80 positive cells was counted within each area. The number of F4/80 positive cells per cubic micron was calculated and adjusted per mm^3^ and the average number for involved and non-involved areas were generated for wild type and knockout samples.

### Implantable tumor models

TC-1 cells (kindly provided by Dr. Ulrich Rodeck) were maintained in RPMI (Corning Life Science) with 1% PenStrep (Gemini Bio-Products) and 10% fetal bovine serum (Benchmark serum, Gemini Bio-Products). TC-1 cells are HPV transformed C57Bl/6 lung epithelium cells (22). For the transplantable tumor model, TC-1 cells were implanted subcutaneously in the shaved right flank of 10-12 week old MCT4^−/−^ and control littermates. 1 × 10^5^ TC-1 cells were injected in 100uL PBS. Tumor area was calculated using the length and width of the tumor (in mm^2^), which was measured using digital calipers. For growth curve studies, animals were sacrificed after 25 days from implantation when the tumor was growing exponentially.

### Blood collection and flow cytometry

Approximately 80 μL of blood was collected in a tube containing 12 μL heparin from the retro-orbital sinus. 40 uL of the collected blood was stained using Zombie Aqua Fixable Viability Kit (BioLegend) and antibodies specific for the monocyte population. Following staining, red blood cell lysis buffer (150 mmol/l NH_4_Cl, 10 mmol/l NaHCO) was added to the samples to isolate leukocytes. CountBright absolute counting beads (ThermoFisher Scientific) were added to the samples immediately prior to analysis. Samples were run on the BD LSRFortessa flow cytometer (BD Biosciences) and data was analyzed using FlowJo Version 10.1 (Treestar).

### Western blot analysis

Cells and tissues were lysed in RIPA buffer (ThermoFisher Scientific) containing protease inhibitor cocktail (ThermoFisher Scientific). Samples were centrifuged and supernatant collected for protein analysis. After protein quantification with the BCA method, samples were separated using Bis-Tris NuPage precast gels (ThermoFisher Scientific) and transferred to PVDF membranes. After blocking with 5% nonfat milk for 1 hour, primary antibodies were incubated overnight at 4°C. Membranes were washed in TBS-tween buffer and incubated in HRP conjugated secondary antibodies for 1h at room temperature. Blots were developed using SuperSignal West Dura ECL substrate (ThermoFisher Scientific) and imaged using FluorChem M ProteinSimple imager.

### Data analysis

All data are presented as mean ± standard error. Data were analyzed with GraphPad Prism v7 (GraphPad software) or R v3.5.0 (R-project.org). Unless specified differently, two-tailed-t test or ANOVA were performed to analyze the mean difference between groups. For correlation analysis Fisher's exact test was used. Data reported in **Figure 6M** data were analyzed using a robust linear mixed effect model with random effect of mice and fixed effect of time, genotype and area. Statistical significance was defined as *p*-value < 0.05.

## Results

### MCT4 expression in human oral squamous cell carcinoma

**Figure 1:**
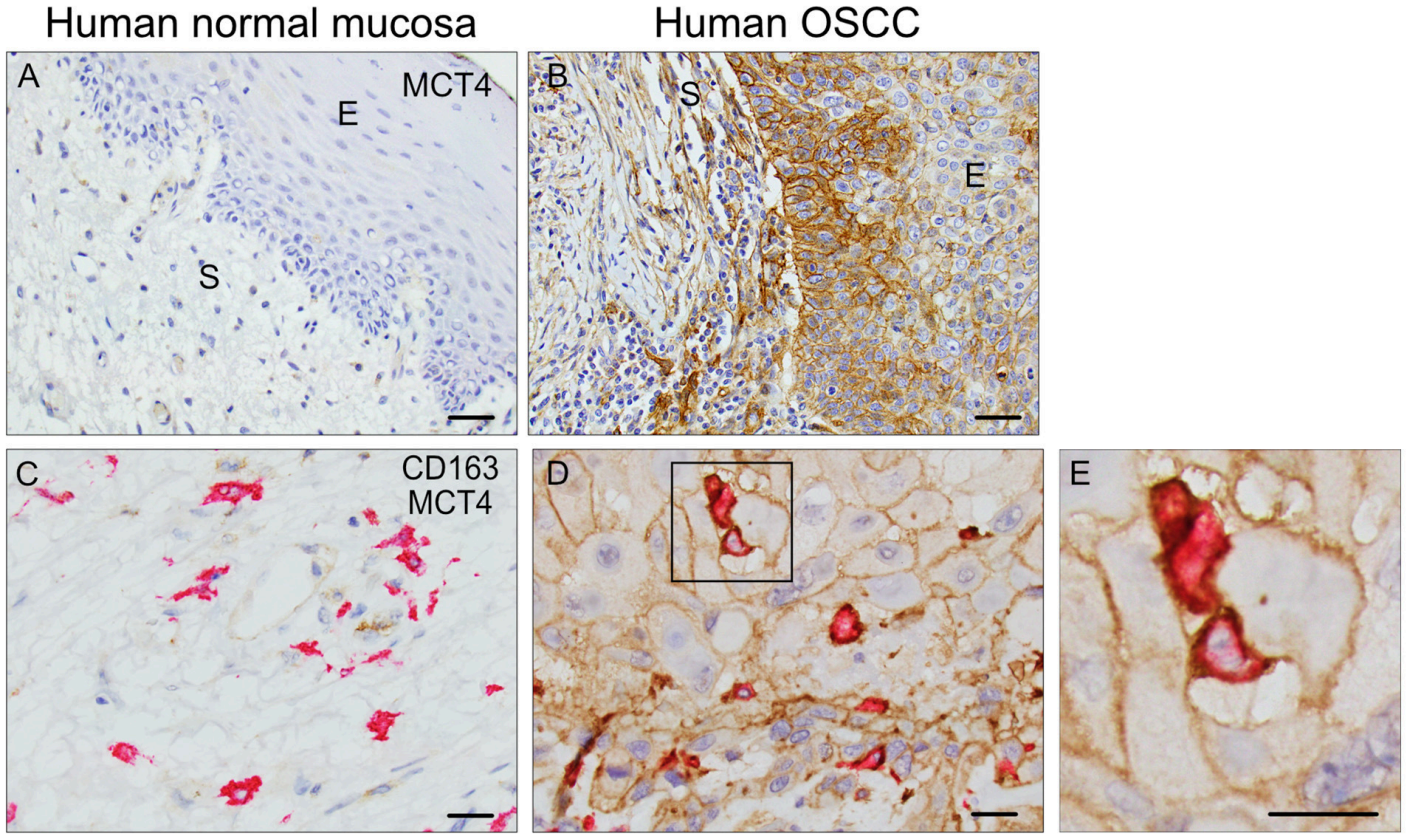
MCT4 is expressed in Human OSCC: Single MCT4 **(A,B)** and dual MCT4/CD163 **(C–E)** immunostaining in paraffin embedded samples of human OSCC. Area of adjacent normal epithelium in OSCC sample #1 **(A)**, area of squamous cell carcinoma within the same sample #1 **(B)**. MCT4 negative/CD163 positive macrophages in adjacent normal area of oral mucosa in sample #2 **(C)**. MCT4 positive/CD163 positive macrophages in areas of cancer cells within same sample #2 **(D)**. Boxed region in D is showed at higher magnification in E (2.5X). Scale bar: 20 μm. S, stroma; E, epithelium.

MCT4 is expressed in both cancer cell and stromal compartments in human OSCC and is typically not present in normal squamous epithelia (Figures 1A,B). In the present study, MCT4 expression was further evaluated in macrophages identified by the marker CD163 in nine cases of HNSCC. The demographic and pathological characteristics of these cases are summarized in Table 1. Paraffin sections were labeled with antibodies to CD163 and MCT4 simultaneously (Figures 1C–E) and the percentage of macrophages positive for MCT4 was evaluated in a blinded fashion by two independent observers (Table 2). CD163-positive macrophages within cancer cell nests or in the immediate adjacent stroma were evaluated at high power and compared to macrophages in normal or non-involved or control areas distant from cancer cell sites. MCT4 staining in macrophages was scored as positive in fields that contained 80% or greater double positive cells. Six of nine cases had macrophages positive for CD163 that were also positive for MCT4 (66.7%). When CD163-positive macrophages were evaluated in non-involved or control areas that were distant and free from the presence of cancer cells, all cases were negative (*p* < 0.01).

**Table 1:** Demographics and Pathologic characteristics.

Mean age (range)	64.9 (50–82)
Gender	6 male/3 females
Tobacco use (>10 pack/year)	6 (67%)
p16 positive	1 (11%)
**SUBSITE**	
Oral cavity	7 (78%)
Oral tongue	2
Floor of mouth	3
Gingiva	2
Oropharynx	2 (22%)
Base of tongue	1
Tonsil	1
**T STAGE**	
T1	1 (1%)
T2	3 (33%)
T4a	5 (56%)
**N STAGE**	
N0	5 (56%)
N2b	3 (33%)
N2c	1 (11%)
**PATHOLOGICAL MARKERS**	
ECE	2 (22%)
Positive margins	2 (22%)
PNI	4 (44%)
LVI	2 (22%)
**DIFFERENTIATION**	
Well	0
Moderate	8 (89%)
Poor	1 (11%)

**Table 2:** HNSCC MCT4 Expression in CD163-positive macrophages.

**Site**	**MCT-4 Positive**	**MCT-4 Negative**	**Total**
Tumor	6	3	9
Control	0	8	8
Total	6	11	17

*Number of samples with CD163 stained macrophages positive or negative for MCT4 in human HNSCC (n = 9). Tumor site is intra-tumoral or immediate peri-tumoral area; Control is adjacent non-tumor i.e., subepithelial stroma or non-involved muscle; Positive is >80% dual positive within field*.

### MCT4^−/−^ mouse model for oral carcinoma development

**Figure 2:**
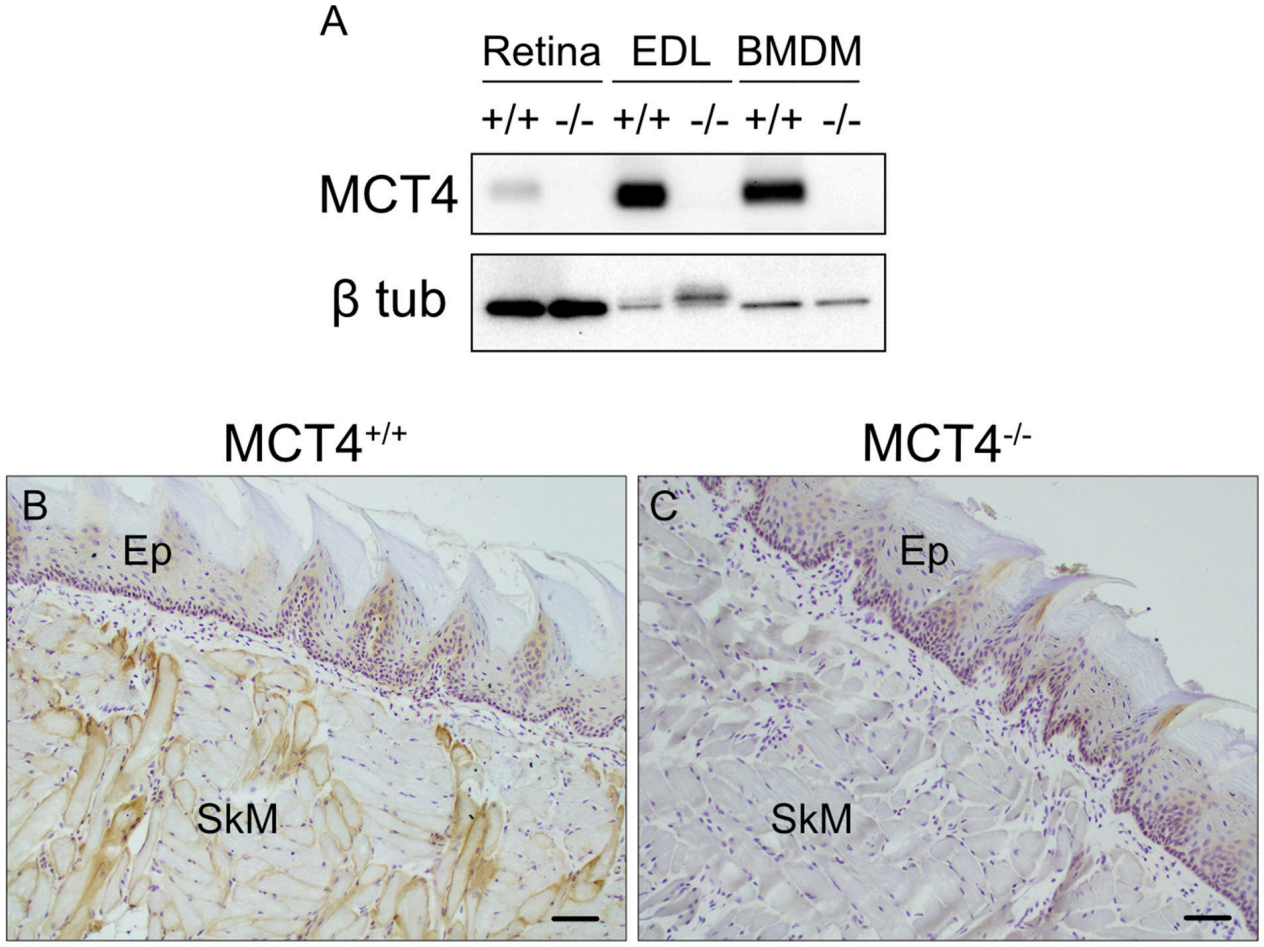
Characterization of MCT4^−/−^ animals. Western blot of detergent lysates prepared from retinas, extensor digitorum longus (EDL) muscle, and LPS activated bone marrow derived macrophages (BMDM) from wild type (+/+) and MCT4 knock out (-/-) mice **(A)**. Frozen sections of mouse tongue from MCT4 ^(+/+)^ **(B)** and MCT4^−/−^ **(C)** mice labeled with MCT4 antibody. Ep, epithelium; SkM, skeletal muscle. Scale bar, 50 μm.

Based on the strong correlation between MCT4 expression and poor outcomes in OSCC we wanted to develop an *in vivo* animal model to test whether MCT4 was a marker or a driver of aggressive cancer. MCT4^+/−^ mice were purchased from Taconic Biosciences and backcrossed to homozygosity, on a C57BL6/N background. No changes in breeding or growth were observed in these mice. Figure 2A is a representative Western blot showing that MCT4 was detected in lysates from retina, skeletal muscle and activated macrophages from wild type but not in MCT4^−/−^ mice (Figure 2A). Frozen sections of wild type and MCT4^−/−^ tongues from animals not exposed to 4NQO were labeled with MCT4 antibody. MCT4 was not present in the mucosa but was detected in the sarcolemma of the skeletal muscle fibers (Figures 2B,C). This pattern of MCT4 expression was identical to that of human oral mucosa (Figure 1A).

### Administration of 4NQO induces more aggressive oral squamous carcinoma in wild type animals

**Figure 3:**
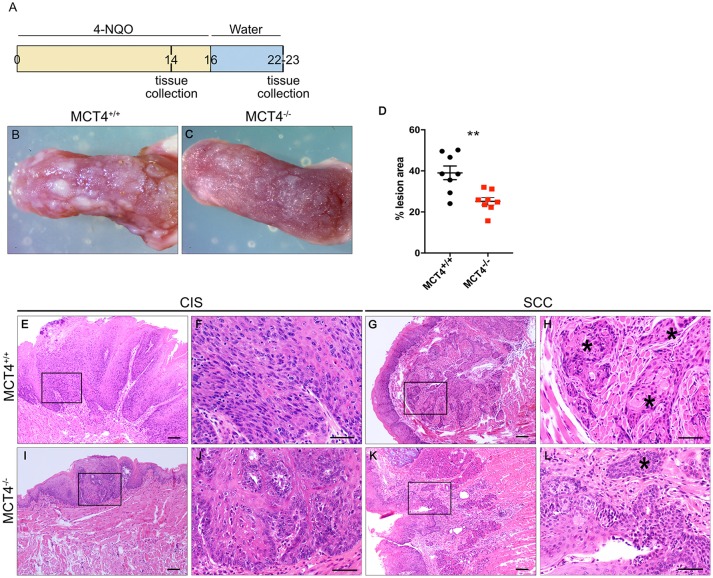
Reduced invasive oral squamous cell carcinoma in MCT4^−/−^ mice after 4NQO: Study design: 12 week old mice were treated for 16 weeks with 4NQO (50μg/ml) in the drinking water, followed by 7 weeks of plain water. Mice were sacrificed after 14 weeks of treatment or at 23 weeks **(A)**. Photographs of wild type **(B)** and MCT4^−/−^ **(C)** tongues after 23 weeks of treatment. Quantification of superficial lesions expressed as percentage of total dorsal tongue surface area (*n* = 8 per group), **p < 0.01 **(D)**. Representative H&E stained paraffin sections of tongues from wild type and MCT4^−/−^ animals treated with 4NQO and sacrificed at 23 weeks **(E–L)** CIS, carcinoma-*in-situ*; SCC, invasive squamous cell carcinoma. F, H, J and L are higher magnifications of E, G, I and K respectively. *Indicates invasive SCC. Scale bar: 100μm **(F,H,J, L)** or 50μm **(E,G,I,K)**.

Treatment of mice with 4NQO in the drinking water is a well-established model for induction of OSCC (23). To understand the contribution of MCT4 to the development and progression of oral squamous cell carcinoma (OSCC), MCT4^−/−^ mice and control littermates were treated for 16 weeks with 50 μg/ml 4NQO in the drinking water, followed by 7 weeks of pure water (Figure 3A). After 23 weeks of treatment, the animals were euthanized and the tongues excised and assessed for cancerous lesions as described in materials and methods. As reported in other studies using 4NQO, during the treatment with the carcinogen the mice lost up to 20% of the initial body weight, but the change was not significantly different between the wild type and the MCT4^−/−^ group. Macroscopic examination of the tongues showed that wild type animals developed more prominent and numerous lesions compared to MCT4^−/−^ animals (Figures 3B,C). Quantification of the area occupied by the white lesions on the dorsal surface of the tongues showed that the wild type animals had 39% of the surface covered by lesions while the MCT4^−/−^ animals had only 25% (*p* < 0.05) (Figure 3D). The tongues of the 23 week 4NQO treated wild type and MCT4^−/−^ mice were paraffin embedded and sectioned. Microscopic observation of H&E-stained sections revealed epithelial changes that ranged from mild to severe dysplasia and carcinoma *in situ* (CIS; Figures 3E,F,I,J) to invasive oral squamous cell carcinoma (OSCC; Figures 3G,H,K,L). There was nuclear enlargement and hyperchromasia that was restricted to the basal layer in areas of mild dysplasia as well as cytologic atypia that involved the entire epithelial thickness, with associated acanthosis and hyperparakeratosis in areas of severe dysplasia and CIS (Figure 3). All animals showed evidence of CIS, however, in MCT4^−/−^ the pathological features of CIS were qualitatively less pronounced, with fewer areas of epithelial involvement (Figures 3E,F,I,J). MCT4^−/−^ mice had fewer invasive lesions (>1 mm depth of invasion), with 2 of 12 knockout mice (16.7%) having invasive SCC compared to 8 of 13 wild type animals with invasive SCC (61.5%; *p* < 0.05) (Figures 3G,H,K,L). However, MCT4^−/−^ mice did show limited micro-invasive features with involvement of less than 1mm depth in 4 of 12 animals (33.3%), compared to 2 of 13 wild type animals (15.4%) but this was not statistically significantly different. The number of CIS, invasive SCC and micro-invasive SCC are summarized in Table 3.

**Table 3:** Histological findings in 23 weeks 4NQO treated wild type and MCT4^−/−^ tongues.

	**wild type**	**MCT4^−/−^**
CIS	13	12
Micro invasive SCC	2	4
Invasive SCC	8	2

*CIS, carcinoma in situ; SCC, squamous cell carcinoma*.

### MCT4 is expressed at an early stage of disease

**Figure 4:**
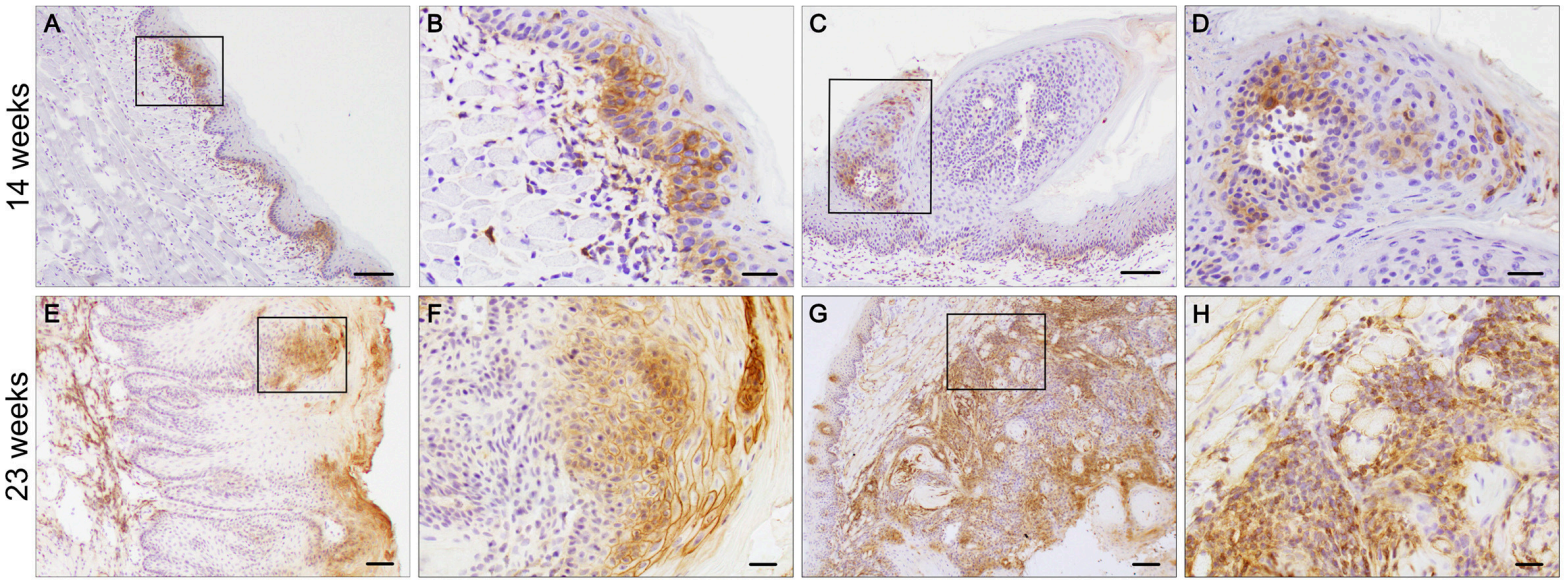
MCT4 is expressed at different stages of oral cancer. Frozen sections of tongues from 4NQO treated wild type mice. MCT4 staining is detected in the basal and suprabasal epithelial layers and in the stroma (**A**, magnified in **B**), in papilloma (**C**, magnified in **D**) after 14 weeks of treatment; and in the upper squamous epithelium of carcinoma *in situ* (**E**, magnified in **F**) and in both the epithelium and stroma of invasive cancer (**G**, magnified in **H**) at the 23 weeks timepoint. Scale bar: 100 μm **(A**,**C**,**E**,**G)** or 20 μm **(B**,**D**,**F**,**H)**.

4NQO treated animals developed a complex range of changes in the epithelium that allowed us to study changes in MCT4 expression during the tumorigenic process. To understand the importance of MCT4 in the development of oral cancer, we sacrificed the animals after 14 weeks of 4NQO treatment when early visible changes could be identified on the surface of the tongues (24). Immunohistochemistry of frozen section of the tongues was performed with MCT4 antibody. Focal expression of MCT4 was detected in the epithelial cells of the stratum basalis as well as in small foci extending into the stratum spinosum (Figures 4A–D). Additionally, in areas where there was epithelial MCT4 expression, we also detected an increase in MCT4 positive cells in the adjacent stroma (Figure 4B). Immunostaining of frozen sections of tongue tissues after 23 weeks showed that with progression of OSCC, the expression of MCT4 persisted and was primarily detected in foci within the upper layers of the epithelium (Figures 4E,F) and often in CIS lesions having a papillary morphology (papillary carcinoma *in-situ*). In regions of invasive cancer, MCT4 was detected in a more widespread pattern throughout the tumor (Figures 4G,H) and was present on transformed epithelial cells and also in adjacent stromal cells within lesional areas.

### Increased expression of MCT1 in OSCC in wild type and MCT4 knock out mice

**Figure 5:**
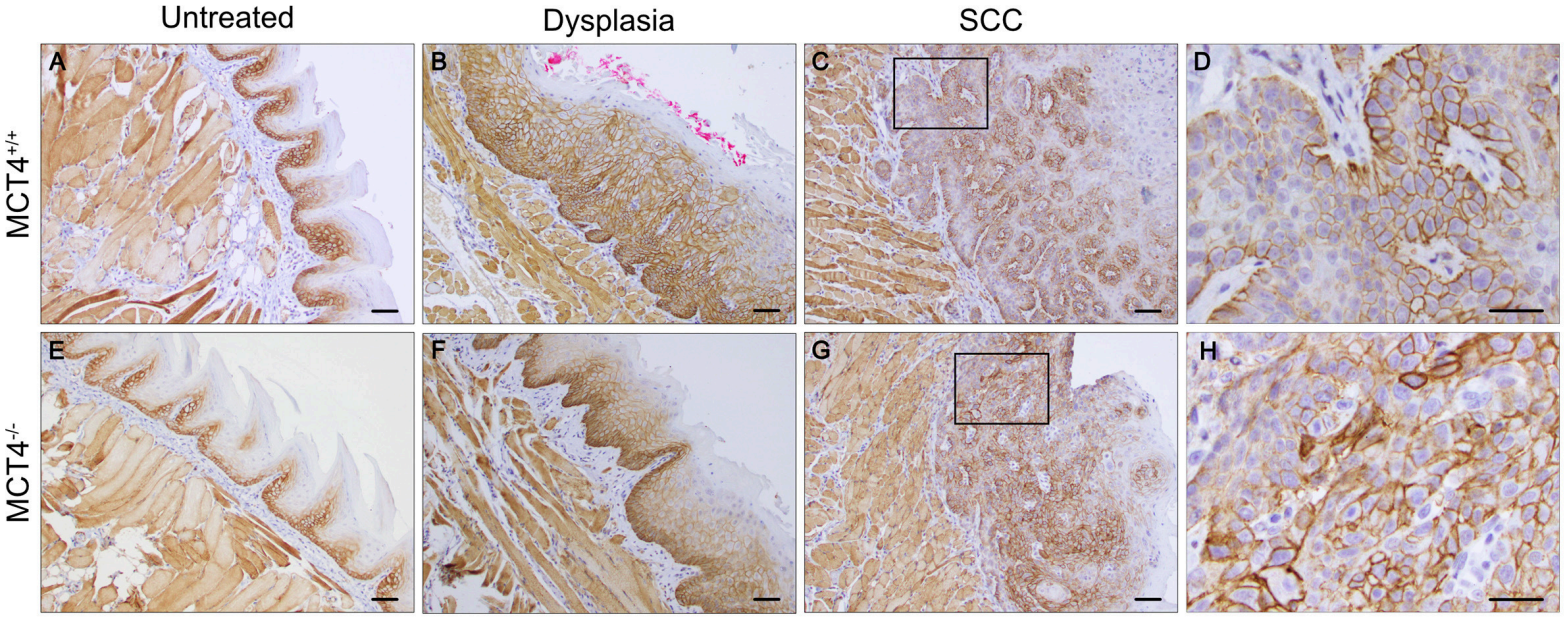
MCT1 expression is altered in 4NQO treated wild type and MCT4^−/−^ animals. IHC staining of paraffin sections of tongues from untreated and 23 weeks 4NQO treated wild type and MCT4^−/−^ mice. MCT1 expression in normal tongue tissue is restricted to the basal cell layer of the epithelium in both wild type and MCT4^−/−^ mice **(A**,**E)**. After 4NQO treatment, MCT1 extends into the suprabasal region of dysplastic lesions **(B,F)**, and is irregularly expressed in areas at the leading edge of invasive cancer **(C,D,G,H)**. Scale bar: 50μm or 20 μm **(D,H)**.

We investigated the expression of MCT1 in wild type and MCT4^−/−^ mice. In tongues from wild type and MCT4^−/−^ untreated mice, MCT1 was primarily restricted to the stratum basalis with faint labeling in the lower layers of the stratum spinosum (Figures 5A,E). This pattern of expression also parallels the MCT1 expression observed in normal human oral squamous epithelium (5, 25). Note that skeletal muscle fibers stain positively for MCT1 as shown in the figure and serve as a positive control. After 4NQO treatment, the expression of MCT1 increased in focal dysplastic areas of the tongues of both wild type and MCT4^−/−^ animals and could be detected in the upper layers of the epithelium (Figures 5B,F). However, in the more aggressive invasive SCC lesions, MCT1 expression became irregular and was confined to groups of cells with an epithelial morphology (Figures 5C,D,G,H). Overall the changes in MCT1 expression patterns were similar in both wild type and MCT4^−/−^ tongues and was not consistent with any compensatory expression due to the absence of MCT4 in MCT4^−/−^ mice.

### MCT4 positive macrophages are present in the stroma

**Figure 6:**
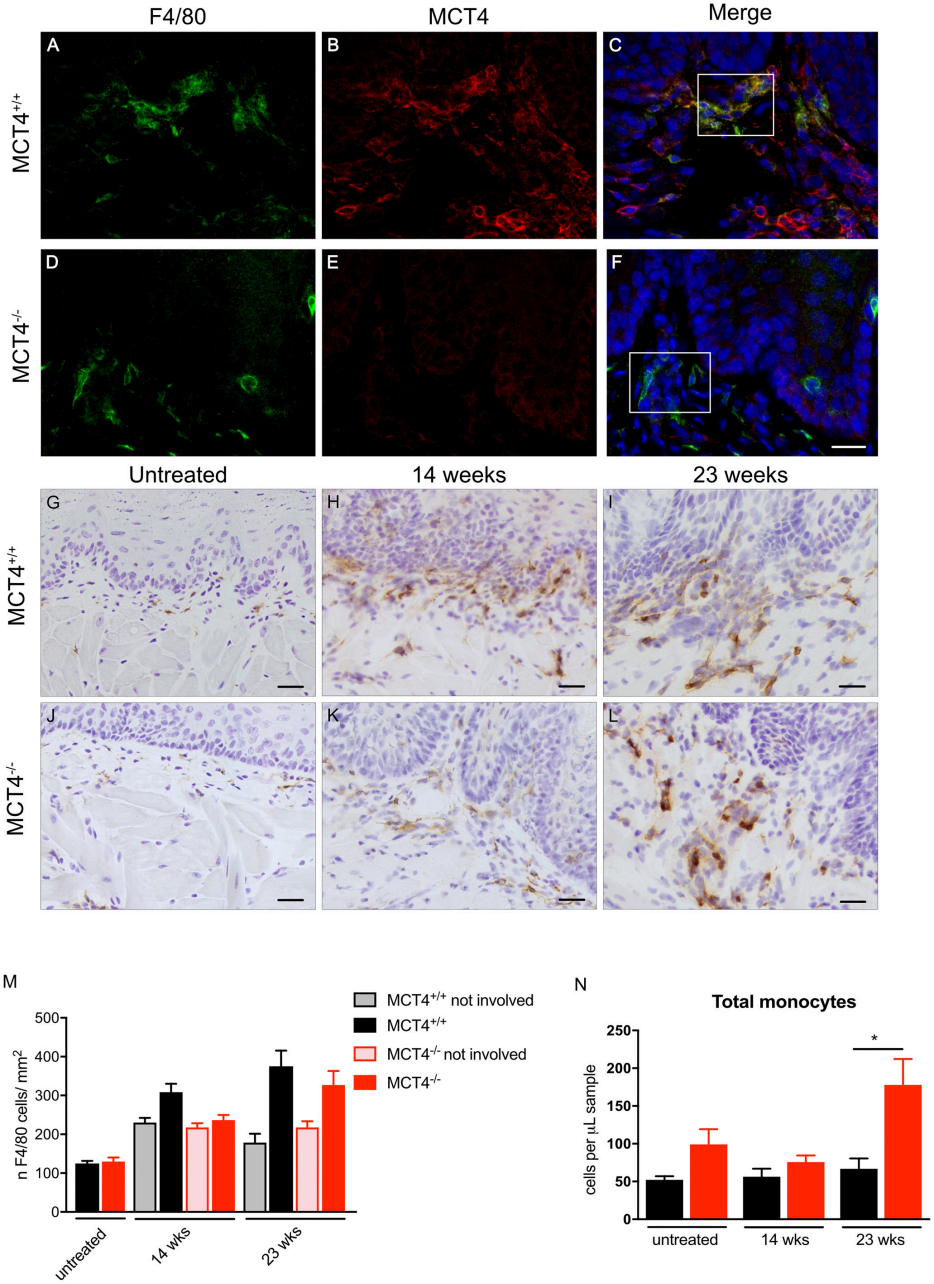
Evaluation of peri-lesional macrophages in wild type and MCT4^−/−^ tumors. Immunofluorescence staining for F4/80 (green) and MCT4 (red) and dapi nuclear staining (blue) in wild type and MCT4^−/−^ tongue frozen sections at 23 weeks of 4NQO treatment **(A–F)** scale bar: 20μm. F4/80 staining of frozen sections in untreated tongues and at 14 and 23 weeks 4NQO treatment in wild type and MCT4^−/−^ animals **(G–L)** Scale bar: 20 μm. Quantification of F4/80 positive macrophages in tongue sections. Involved areas represent regions identified by nuclear atypia and/or other signs of epithelial transformation, whereas non-involved areas did not display these features within the same section (*n* = 3 for untreated, *n* = 8 for 14 weeks, *n* = 4 for 23 weeks per group) **(M)**. Quantification of total monocytes **(N)** in peripheral blood (*n* = 5–6 per group). *P < 0.05.

In the human OSCC, MCT4 positive macrophages were found within the lesions and in the adjacent stroma. Therefore, we investigated the presence of MCT4 positive macrophages in the 4NQO treated animals. Macrophages identified by F4/80 were positive for MCT4 in wild type mice at both 14 weeks (data not shown) and 23 weeks (Figures 6A–C). While F4/80 positive cells were detected in the mucosa of MCT4^−/−^ mice, they were not co-labeled with MCT4 (Figures 6D–F). To determine whether there was a difference in macrophage number between wild type and MCT4^−/−^ mice, tongue sections from untreated, and 14 and 23 week 4NQO-treated mice were stained with F4/80 by immunohistochemistry (Figures 6G–L) and the number of positive cells was quantified as described in Materials and Methods. In normal untreated wild type and MCT4^−/−^ tongues the numbers of F4/80 positive macrophages were not significantly different (Figure 6M), with both groups having similar baseline numbers of macrophages. After 14 weeks of 4NQO treatment the number of macrophages present in the involved regions (perilesional areas) of wild type animals was 23% greater than in the MCT4^−/−^ animals (*p* < 0.05). By 23 weeks, the number of macrophages in the MCT4^−/−^ affected areas was similar to the wild type. We also evaluated the number of macrophages in non-involved areas, (not lesional) and they were similar in both wild type and MCT4^−/−^ animals exposed to 4NQO, although the numbers in the lesional areas were significantly increased compared to the non-involved areas (average difference 35.3, *p* < 0.03). This indicates that the increase in macrophages is due to the presence of cancer cells and not because of a general inflammatory response to the 4NQO.

We also analyzed the levels of circulating monocytes in the 4NQO treated animals to determine if the differences in macrophage number in the lesion was due to a defect in recruitment and mobilization. The total number of monocytes in circulation was higher in the untreated MCT4^−/−^ animals, but the difference was not statistically significant. At 23 weeks after 4NQO treatment, the number of circulating monocytes in the MCT4^−/−^ animals was 2.6 times higher than the wild type (*p* < 0.05), suggesting a defect in the recruitment of macrophages to the tumors.

### MCT4 in syngeneic tumors is sufficient to drive cancer growth

**Figure 7:**
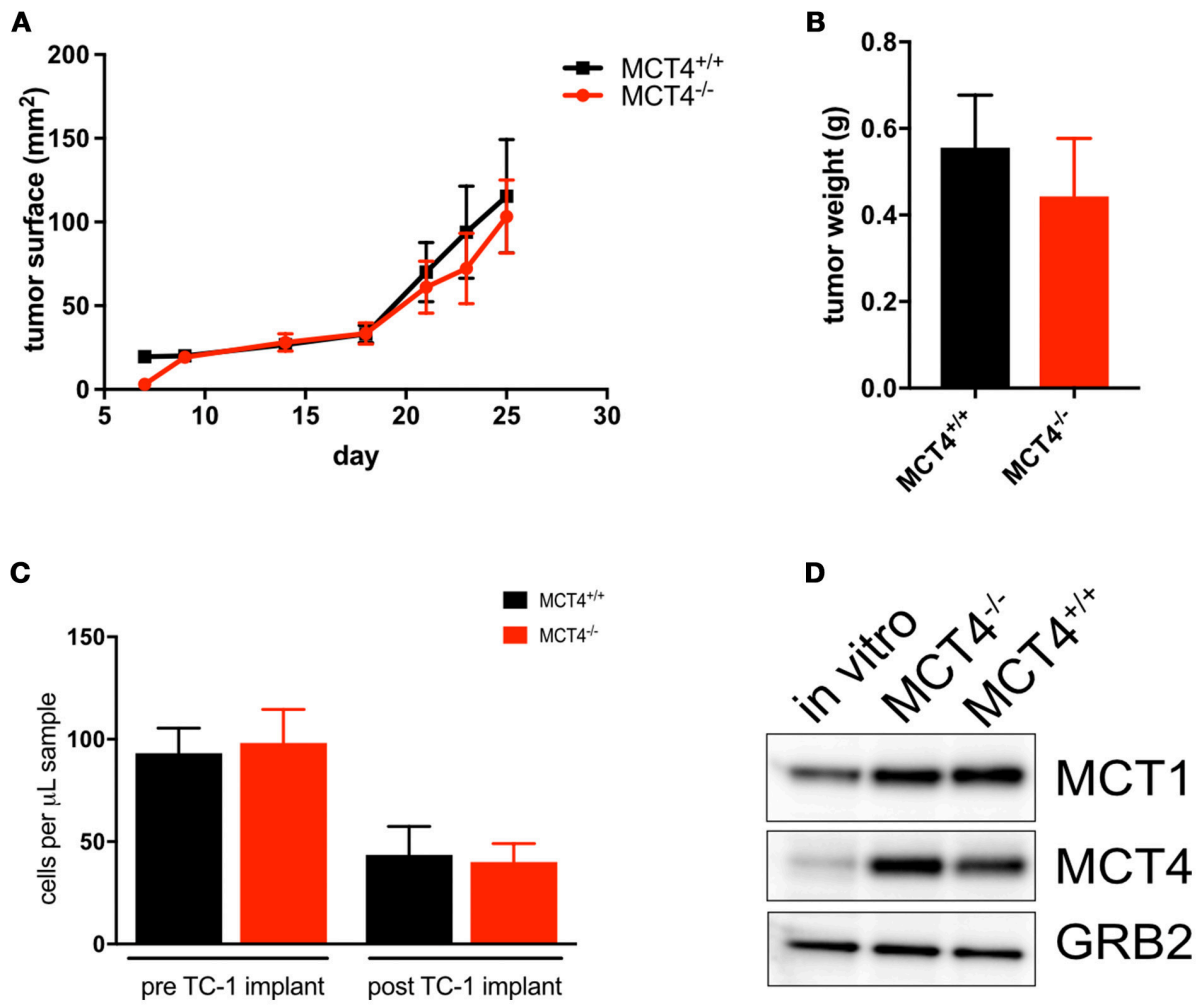
MCT4 expression in syngeneic tumor cells is sufficient for tumor growth. Growth curve of TC-1 tumors in MCT4^−/−^ and wild type mice (*n* = 10 per group) **(A)** and average tumor weight at sacrifice **(B)**. Quantification of total monocytes in peripheral blood of wild type and MCT4^−/−^ animals before and after transplantation (*n* = 5 per group) **(C)**. Western blot analysis of lysates prepared from TC-1 cancer cells *in vitro* and TC-1 tumors grown in MCT4^−/−^ and wild type mice for MCT1 and MCT4 using GRB2 as a loading control **(D)**.

To investigate the contribution of MCT4 expressed in tumor stroma to the development and progression of the OSCC, TC-1 cells, derived from C57Bl/6 lung carcinoma were implanted into the flanks of MCT4^−/−^ mice and control littermates. Surprisingly the tumor growth was independent of the host genotype with similar tumor sizes in wild type and MCT4^−/−^ animals (Figures 7A–B). Additionally, there was no difference in the number of circulating monocytes between wild type and MCT4^−/−^ mice (Figure 7C). Since we were perplexed by this finding we examined the expression of MCT1 and MCT4 in TC-1 cells cultured *in vitro* and from the syngeneic tumors. We found that the level of MCT4 in TC-1 tumors was increased significantly compared to the basal expression levels detected in lysates of cultured TC-1 cells (Figures 7D). The result suggests that MCT4 expression by the epithelial cells is sufficient to support tumor growth in this syngeneic model.

## Discussion

MCT4 expression in cancers such as HNSCC, breast cancer, melanoma, and hepatocellular carcinoma has been associated with a poor prognosis (5, 26–28). In patient tumors, MCT4 has been detected in cancer cells, cancer associated fibroblasts (CAFs) as well as tumor associated macrophages (TAMs) where it supports tumor growth (17), angiogenesis (29), and metastasis (30) (Figure 1). In the current study, we used the well-established 4NQO model of carcinogenesis to induce OSCC in wild type and MCT4^−/−^ mice, with the goal of determining whether MCT4 is a driver or marker of OSCC progression. Our findings show that in the absence of MCT4, tumor burden, and invasiveness is reduced.

Growth and metastasis of cancer cells is supported by CAFs and TAMs present in the tumor ecosystem. The use of 4NQO in the drinking water to induce OSCC has several advantages over syngeneic models. Chemical induction of OSCC results in tumor formation in the normal ecosystem so the development and progression to invasive cancer more closely mimics what is seen in human patients (23). Models using transplantable OSCC cell lines do not recapitulate the epithelial and stromal interactions since the cells are injected into the flank of the mouse between the hypodermis and skeletal muscles. This region lacks the capillary bed and stroma found in the lamina propria of epithelial tumors. The 4NQO model recapitulates histological features and metabolic reprogramming observed in patient samples of OSCC and thus provides a valuable model to investigate *in vivo* the contribution of MCT4 to the development and progression of OSCC.

In 4NQO treated animals, the cancer originates from the highly proliferative cells found in the basal cell layer of the stratified squamous epithelium covering the tongue (31). 4NQO treatment causes mutagenesis and oxidative stress in these cells, which in turn translates in the dysregulation of key transcription factors such as NF-kB (32) and Hif1α (33). The cross-talk between the two transcription factors leads to the activation of glycolysis (34, 35) through transcription of glycolytic genes including MCT4 (36), glucose transporter 1 (GLUT1), hexokinase (HK), lactate dehydrogenase A (LDHA), and genes regulating cell proliferative pathways. In the current study, MCT4 was not detected in epithelium or stroma of the untreated tongue but was detected focally in the basal and suprabasal layers of the epithelium in tongues of wild type mice treated with 4NQO (Figures 4A,B). The MCT4^−/−^ mice treated with 4NQO also developed dysplastic lesions and carcinomas *in situ* (CIS) similarly to the wild type mice, demonstrating that MCT4 is not necessary for the induction of non-invasive OSCC. Our findings indicate that the early increase in MCT4 expression results from a global reprogramming of the epithelial cells undergoing oncogenic transformation. When we analyzed MCT4 expression in more severe and extensive lesions such as carcinomas *in situ*, we found that MCT4 was often expressed in a central compartment of the lesions (Figures 4E,F), which may correspond with hypoxic areas. This centralized pattern of expression replicates what has been documented in human OSCC (5). Future studies will be needed to confirm if this pattern correlates with regions of hypoxia. Large *in situ* lesions were not found in tongues from MCT4^−/−^ mice treated with 4NQO since these epithelial cells cannot shift their metabolism toward a hyperglycolytic state. It has been shown that inhibition of the lactate transport has a negative effect on cell proliferation (17, 37). The intracellular accumulation of lactate slows the glycolytic flux by directly inhibiting hexokinase and phosphofructokinase (38) and by decreasing the NAD^+^/NADH ratio. In addition, the decrease in glycolytic intermediates affects the production of other metabolites necessary for cell proliferation. The inhibition of MCT4 also leads to the acidification of the cytosol, which can inhibit cell proliferation through the mTOR pathway (39). In sum, future studies will need to be conducted to determine the mechanism by which loss of MCT4 reduces tumor aggressiveness.

The current study showed that MCT4^−/−^ mice developed fewer and smaller invasive carcinomas, more often they developed micro-invasive lesions. Additional studies are required to establish the contribution of MCT4 to microinvasive oral squamous cell tumors. The data suggest that the lack of MCT4 prevents the progression of early microinvasive lesions to full invasive, arresting the tumors in a less aggressive form. In the wild type invasive lesions, MCT4 was expressed in both epithelial cells and in stroma (Figures 4G,H). In wild type mice expression of MCT4 facilitates lactate efflux and acidification of the microenvironment promoting invasiveness and metastasis. We and others have previously shown that MCT4 is important for cancer cell migration through interaction with beta 1 integrin (6, 40, 41) and it has been shown that lactate in the microenvironment induce angiogenesis (42), contributing to the progression of the disease.

We have shown that in human samples of HNSCC, CD163 positive macrophages also express MCT4 (Figures 1D,E). In the 4NQO treated animals we found macrophages in the peri-lesional areas as early as 14 weeks of treatment and these F4/80 positive cells expressed MCT4 (Figures 6A–C). In the MCT4^−/−^ animals, the number of macrophages noted in the tumor microenvironment is lower compared to the wild type (Figure 6M) suggesting that MCT4 in either one of the compartments, or in both of them, has an effect on the macrophage population. MCT4 is usually associated with a higher glycolytic flux, therefore TAMs may have a glycolytic metabolism as suggested by Liu et al. (43). It has also been reported that bone marrow derived macrophages (BMDM) knock down of MCT4 reduced the production of cytokines after LPS stimulation (12), indicating that the F4/80 cells in the tumor microenvironment of the MCT4^−/−^ animals may be compromised. Macrophages are recruited to the tumor invasive edge by chemoattractants released by the cancer cells. The recruited immune cells promote the migration and invasion of the cancer by secreting factors for the remodeling of the extracellular matrix (44). The observation that fewer macrophages were present in the stroma of the MCT4^−/−^ tongues suggest that there was a failure of the tumor to release sufficient levels of chemokines and cytokines required to recruit monocytes from circulation. This conclusion was supported by our analysis of the peripheral blood from 4NQO treated MCT4^−/−^ mice which showed an increase in total monocytes in circulation (Figure 6N). The circulating monocytes were the same in wild type and MCT4^−/−^ mice after TC-1 syngeneic tumors implantation and the tumors expressed MCT4 independently of the genotype of the host. This supports the idea that MCT4 expression in the epithelium is necessary for the recruitment of macrophages to the tumor microenvironment from the circulation (Figure 7) but future studies will need to determine the contribution of MCT4 expression in TC-1 cells to syngeneic tumor growth.

MCT1 and MCT4 require the accessory protein CD147 for maturation and trafficking to the plasma membrane. CD147 is constitutively expressed and is stabilized through its interaction with MCT1 and MCT4 (45). Consistently, we and others have used *in vitro* and *in vivo* techniques to show that ablation of any of these proteins (MCTs or CD147) causes the degradation of the entire complex. Conversely, the increase in MCT1 and/or MCT4 in OSCC results in the obligatory increase in CD147. In 4NQO-treated wild type and MCT4^−/−^ mice, MCT1 expression increased in dysplastic areas. The invasive lesions also showed persistent expression of MCT1 (Figure 5). It has been reported that increased expression of CD147 (also known as EMMPRIN and basigin) is associated with poor outcomes in OSCC (8). The use of drugs targeting CD147 in cancer has been explored (39) and the use of anti-CD147 antibodies resulted in reduced growth of OSCC xenografts (40). Silencing CD147 or using drugs that target CD147 impact the expression or activity of MCT1 and MCT4, supporting the idea that targeting these transporters in patients with OSCC could slow the development and progression of the disease.

This study showed that MCT4 is an early marker of OSCC and that MCT4^−/−^ mice had fewer lesions and less invasive cancer. MCT4 could provide a viable target for drug therapy to slow progression and aggressiveness in OSCC.

## Ethics statement

**Human study:** The study was carried in accordance with the recommendation of Institutional review board (IRB) at Thomas Jefferson University, with written informed consent from all subjects. All subjects gave written informed consent in accordance with the Declaration of Helsinki. The protocol was approved by the IRB at Thomas Jefferson University.

**Animal study:** The study was carried out in accordance with the recommendations of the IACUC of Thomas Jefferson University. The protocol was approved by The IACUC at Thomas Jefferson University.

## Author contributions

SB contributed to the design of the experiments, performed all animal experiments, contributed to histological staining and analysis of tissue, analysis of data, and writing the manuscript. DW-M contributed to the design of the experiments, performed immunohistochemistry and immunofluorescence, performed animal experiments, analyzed tissue, writing of manuscript. NW performed the syngeneic tumor injections and analysis of peripheral blood. MT performed histopathological analysis. JC collected human samples, contributed to discussion. TZ performed data analysis. CS contributed to experimental design and discussion. UM-O contributed to experimental design, data analysis and writing the manuscript. NP contributed to experimental design, data analysis, and writing the manuscript.

### Conflict of interest statement

The authors declare that the research was conducted in the absence of any commercial or financial relationships that could be construed as a potential conflict of interest.
